# Integrated Transcriptome and Targeted Metabolome for Resolving Flavonoid Biosynthesis in Figs (*Ficus carica Linn*.)

**DOI:** 10.3390/biology14020184

**Published:** 2025-02-11

**Authors:** Junting Sun, Hadir Yishake, Ming Wang, Hao Zhang, Jie Yan

**Affiliations:** 1 College of Life Sciences, Shihezi University, Shihezi 832000, China; j15299814146@163.com; 2 Xinjiang Academy of Forestry Sciences, Urumqi 830000, China; xjkde1997@sina.com (H.Y.); 15299922603@163.com (M.W.)

**Keywords:** figs, flavonoid, biosynthesis, transcriptome, metabolome

## Abstract

Figs are an edible and medicinal plant rich in polyphenols and flavonoids with unique pharmacological effects. However, the mechanism of flavonoid synthesis in figs is not clear. In this study, fig fruits of six varieties were collected for RNA sequencing and UPLC-MS data collection. The results showed that a total of 39 differential metabolites were identified by targeted metabolomics, and their contents were determined by UPLC-MS. The clustered heat map analysis showed that most of the differential metabolites were highly accumulated in BRD and FY. A total of 62 flavonoid biosynthesis pathway genes were identified by transcriptome analysis, and *FcCHS*, *FcCHI*, *FcFLS*, *FcCYP*, and *FcDFR* were the key genes identified for the accumulation of flavonoids and flavonols in the dark-colored varieties. In addition, a total of 1671 transcription factor genes, mainly MYBs, bHLHs, and AP2/ERFs, were identified. This study will enrich the transcriptomic data of figs and provide some help in resolving the synthesis mechanism of fig flavonoids.

## Simple Summary

This study explored the synthesis mechanism of flavonoids in fig fruits by analyzing six different varieties using RNA sequencing and metabolomics (UPLC-MS). The research identified 39 flavonoid-related metabolites and 62 genes linked to flavonoid biosynthesis, with key genes like “*FcCHS*”, “*FcCHI*”, and “*FcFLS*” playing a role in higher flavonoid levels in dark-colored figs. Additionally, 1,671 transcription factors (e.g., MYBs, bHLHs) were found, potentially regulating flavonoid production. The findings provide new insights into the metabolic pathways of flavonoid synthesis in figs, aiding future research and applications in plant medicine and nutrition.

## 1. Introduction

Fig (*Ficus carica* L.) is a subgenus of Ficus in the Moraceae family and is frequently referred to as the “common fig” [1]. Its fruit, leaves, stems, and roots are abundant in carbohydrates, amino acids, unsaturated fatty acids, polyphenols, minerals, and other nutrients, which possess antioxidant, anti-tumor, hypoglycaemic, hypolipidemic, and antibacterial properties [2,3]. Flavonoids are also one of their primary metabolites and have become characteristic substances due to their unique pharmacological effects, which have garnered significant attention in recent years [4,5,6]. Flavonoids are also essential for the antiretroviral effects of plants [7] and their pharmacological effects. Specifically, flavonoids can effectively mitigate the effects of ultraviolet rays on plants, improve their resistance to cold at low temperatures [8], mitigate damage caused by drought stress on plants [9], improve resistance to salt stress [10], and enhance antimicrobial activity [11,12]. Studies on the regulatory mechanisms of flavonoids in other plants are progressively increasing [13], and the mechanism of flavonoid synthesis in *Arabidopsis thaliana* is now more thoroughly investigated. The accumulation of flavonoids in cucumber is regulated by *CsMYB60*, which in turn governs the rind color of ripe fruits [14]. The CHS gene regulates the synthesis of flavonoids in response to light stress [15]. *PpbZIP44* governs the accumulation of amino acids and flavonoids in pear fruit and carbohydrate metabolism [16]. *DzMYB1*, a novel MYB transcription factor in durian, regulates the fruit pulp’s flavonoid biosynthesis. In this study, UPLC-MS will be used to determine the content of flavonoids in figs and to analyze the regulatory mechanism of flavonoid synthesis in figs in conjunction with bi-omics.

The transcriptome is the comprehensive RNA transcripts of organisms and cells at a specific time and location, serving as a critical link between phenotypes and DNA coding information [17]. This information can be used to elucidate the molecular mechanisms of biological processes. The metabolome is an analytical technique that can intuitively reflect the differences in metabolic levels of organisms by conducting a comprehensive study of metabolites downstream of the genome. This study can then be used to postulate on the relevant metabolic pathways and metabolic networks [18]. It offers novel concepts and methodologies. It provides novel approaches and ideas for the identification of plant active metabolites, the analysis of metabolic pathways, and the investigation of plant stress tolerance. Dual-omics and multi-omics analyses are becoming increasingly prevalent due to the complexity of plant metabolic pathways and the diversity of molecular mechanisms of metabolism, which render single-omics analyses incapable of satisfying the requirements of contemporary research [19]. For instance, the mechanism of polyphenol biosynthesis in dandelion was revealed by integrating transcriptomic and metabolomic analyses [20]. The role of phytohormone signaling and lipid metabolism in developing Nitraria sibirica leaves was shown by the combined analysis of transcriptomic and metabolomic data [21]. Integrating the transcriptome and metabolome during the development of quinoa seeds revealed the accumulation of related metabolites and metabolic regulatory networks [22]. The integration of transcriptome and metabolomics analyses suggested that jasmonates may facilitate the development of the Cucumber Green Mottle Mosaic Virus in Bottle Gourd infestation [23].

Most current flavonoid research focuses on extraction, purification, and bioactivity. For instance, fig leaf polysaccharides’ physicochemical properties and bioactivity were investigated using fig leaves as materials, and the results indicated that ultrasound-assisted extraction had the most significant bioactivity effect [24]. Nevertheless, the regulation of flavonoid synthesis in various fig fruit varieties has not been reported. Consequently, the current study opted to conduct a combined transcriptomic and metabolomic analysis to examine flavonoid synthesis in fruits of various varieties. Results of this analysis included identifying a total of 40 metabolites and the expression profiles of genes associated with flavonoid biosynthesis. In addition to enriching the transcriptomic and metabolomic data of figs, this study also investigated the molecular mechanism of flavonoid synthesis in fig fruits at the molecular level, revealed the expression of flavonoid metabolites and biosynthesis pathway genes, and will be beneficial for the selection of fig varieties and flavonoid biosynthesis.

## 2. Materials and Methods

### 2.1. Plant Materials

‘Qing Pi (QP), A134 (MLY), California Black Mission (FY), White Adriatic (BY), B1011 (JZ), and Vidette de Bordeaux (BRD)’ choose from six types of figs that vary greatly in shape, size, and color. Qingpi, introduced from Weihai, Shandong Province, has fruit that is oblate or obconic, the skin is yellowish-green at maturity, the flesh is mauve, the fruit is small, the fruit surface is smooth, and the skin is tough. A134, introduced from California, has ovoid fruit, the skin is golden yellow and shiny, and the flesh is brown or light yellow, slightly hollow, with a sweet taste. California Black Mission originated from Spain and was later introduced to California. The fruit is long and ovoid, the skin is purple-black at maturity, and the flesh is light strawberry color. White Adriatic was introduced from Italy; the fruit is an oblate or inverted cone, the skin is green at maturity, the flesh is red, the fruit is small, the surface is smooth, the skin is tough, and the fruit is large. B1011, introduced from California, USA, has a large fruit that is obovoid, with golden-yellow, glossy skin, obvious ribs, and pinkish-red, hollow flesh. Violette de Bordeaux originated from Spain; the fruit is small, with dark purple skin and bright red flesh at maturity.

These six varieties of figs come from the greenhouse of Shuixigou Fig Plantation in Urumqi County, Urumqi City, China (87°30′22.169″ E, 43°27′52.798″ N). It belongs to the northern hemisphere’s warm, temperate, arid desert climate, rich in light and heat resources, and the soil is sandy loam, suitable for fig cultivation and production. Fig fruits were selected from 5-year-old fig plants, harvested in October 2023, and all were 2 months after fruit set. The fruits of the six varieties were harvested in three biological replicates, and the fruits of the same varieties were mixed together and immediately frozen in liquid nitrogen and stored in a refrigerator at −80 °C for subsequent extraction of RNA and metabolites.

### 2.2. Sample Extraction and Targeted Metabolomic Analysis

Acetonitrile, acetic acid, and methanol were procured from ANPEL. The solvents were all of LC-MS grade. Additionally, ultra-pure water is generated in-house using a Milli-Q water purification system (Millipore, Bedford, MA, USA), with standards, it is level 1 (highest confidence level, standard validation)

The samples were extracted with 0.5 mL of 80 percent methanol (containing 0.2% VC) and ultrasonic vibrations for 30 min. The supernatant was collected after centrifugation at 12,000 rpm for 10 min. The extraction was repeated three times, and the supernatant was collected in a tube. The total weight of the samples was approximately 100 mg [25]. The sample extracts were analyzed using a UPLC–Orbitrap-MS system (UPLC, Vanquish; MS, QE). The following were the analytical conditions: UPLC: column, Waters HSS T3 (50 × 2.1 mm, 1.8 μm); column temperature, 40 °C; flow rate, 0.3 mL/min; injection volume, 2 μL; solvent system, water (0.1% acetic acid), acetonitrile (0.1% acetic acid); gradient program, 90:10 *v*/*v* at 0 min, 90:10 *v*/*v* at 2.0 min, 40:60 *v*/*v* at 6.0 min, 40:60 *v*/*v* at 8.0 min, 90:10 *v*/*v* at 8.1 min, 90:10 *v*/*v* at 12.0 min [26].

The Fullms-ms2 acquisition methods were employed to record HRMS data on a Q Exactive hybrid Q–Orbitrap mass spectrometer (Thermo Fisher Scientific, Waltham, MA, USA) furnished with a heated ESI source. The parameters for the ESI source were established as follows: −2.8 kV spray voltage; 40 arb sheath gas pressure; 10 arb aux gas pressure; 0 arb sweep gas pressure; 320 °C capillary temperature; and 350 °C aux gas heater temperature [27].

Data were collected on the Q-Exactive using Xcalibur 4.1 (Thermo Scientific) and processed using TraceFinder™ 4.1 Clinical (Thermo Scientific). Quantified data were exported to an Excel spreadsheet.

### 2.3. Total RNA Extraction and Transcriptome Sequencing Analysis

The Agilent 2100 bioanalyzer (Agilent, Santa Clara, CA, USA) was employed to accurately detect each sample’s total amount and RNA integrity after total RNA was extracted using a plant RNA purification reagent (Invitrogen, Carlsbad, CA, USA). The mRNA with polyA tails was later enriched by Oligo(dT) magnetic beads, followed by random interruption of the resulting mRNA with divalent cations in the fragmentation buffer. Random oligonucleotides were employed as primers to synthesize the first strand of cDNA in the M-MuLV reverse transcriptase system, which was followed by the degradation of the RNA strand with RNaseH and the synthesis of the second strand of cDNA with dNTPs in the DNA polymerase system. The fragmented mRNA served as the template. The purified double-stranded cDNA was end-repaired, A-tailed, and connected to the sequencing connector. The cDNA was approximately 370,420 bp in length and was screened using AMPureXP beads (Beckman Coulter, Brea, CA, USA). PCR amplification was conducted, and the PCR product was purified again using AMPure XP beads. The library was subsequently obtained. Following the library’s construction, the Qubit2.0 Fluorometer was employed to conduct preliminary quantification, and the library was attenuated to 1.5 ng/μL. After the Agilent 2100 analyzer was used to detect the insert size of the library, the effective concentration of the library was accurately quantified using qRT-PCR. The insert size was as anticipated. The library’s quality was guaranteed by quantification, which indicated that the effective concentration was greater than 1.5 nM. After passing the library examination, different libraries were pooled based on the effective concentration and the target downstream data volume for Illumina sequencing. The 150 bp paired-end reads were generated. The sequenced fragments were converted into sequence data (reads) through CASAVA base recognition of the image data, measured by the high-throughput sequencer. The transcripts underwent Trinity splicing, and the H-Cluster algorithm was employed to generate new clusters. The assembled transcripts were subsequently assessed using tblastn, Augustus, and Hammer. The edgeR (3.22.5) R package was hired to conduct differential expression analysis of the two samples. Significant differential expression was defined as q-values < 0.05 and $\left|log_{2}\left(foldchange\right)\right|$ > 1 [28]. The differential gene sets were subjected to GO functional enrichment analyses, KEGG pathway enrichment analyses, and other analyses using GOseq and KOBAS 2.0 software. The differential gene screening criteria were set to padj < 0.05 and $\left|log_{2}\left(foldchange\right)\right|$ > 1.

### 2.4. Quantitative Real-Time PCR Analyses

Eight genes were selected to analyze the gene expression levels by quantitative fluorescence PCR. Reverse transcription was performed using EasyScript One-Step gDNA Synthesis SuperMix EasyScript (code: AE311-03, TransGen, Beijing, China) according to the instructions, and the primer sequences of the eight candidate genes and the internal reference genes are shown in S1. The qRT-PCR analyses were performed using the LightCycler 480 System (Roche, Basel, Switzerland) and ChamQ Blue Universal SYBR qPCR Master Mix. Three replicates were performed for each fig variety and each qRT-PCR reaction included three technical replicates. The relative expression levels of the candidate genes were calculated using the $2^{-\Delta \Delta Ct}$ method [29].

### 2.5. Statistical Analysis

Building PCA and OPLS-DA modeling analyses using SIMCA 16 software. The K-means analysis and hierarchical cluster analysis were performed using the ComplexHeatMap R package. Annotation of differential metabolites based on the KEGG database. Differential expression analysis was performed using the edgeR (3.22.5) R package. GO functional enrichment analysis of differential gene sets, KEGG pathway enrichment analysis, etc. using GOseq and KOBAS software. The ANOVA test was used in Figure 8.

## 3. Results

### 3.1. PCA and Clustering Heatmap Analyses of Metabolites from Six Varieties of Figs

The flavonoid-targeted metabolomics of the figs of ‘Qing Pi’, ‘A134’, ‘California Black Mission’, and ‘White Adriatic’, as well as the ‘B1011’ and ‘Vidette de Bordeaux’ figs, were analyzed using UPLC-MS. A total of 40 compounds were identified (Table S1).

**Figure 1:**
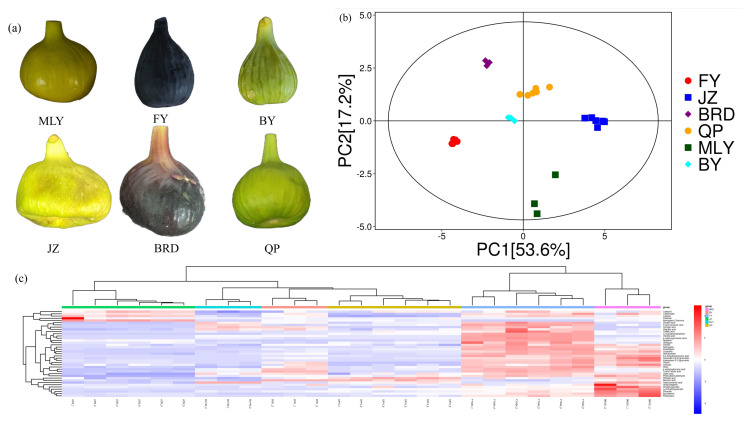
Photos of the 6 varieties of figs (**a**), principal component analysis (PCA) (**b**), and hierarchical clustering heatmap analysis of metabolites identified (**c**).

Principal component analysis (PCA) was employed to analyze similarities and differences between samples within and between groups. The results show that the entire sample lies within the 95% confidence interval, with PC1 and PC2 explaining 53.6% and 17.2% of the total variance (Figure 1b, Table S2). The distribution of samples in the figure shows that different groups of samples are located differently in space. Samples in the same group are more clustered, indicating a higher similarity of the samples within the group; samples between different groups are farther apart, indicating greater differences between groups. Observing the distribution of sample points, it is possible to make a preliminary judgment on the differences and similarities of flavonoid metabolites between different groups of samples. Hierarchical clustering heat maps were employed to cluster and analyze the flavonoid metabolites of QP, MLY, FY, BY, JZ, and BRD. The results showed that there was a clear grouping of the six varieties, and the clustering heat map clustered the different metabolites together, suggesting that these metabolites may have a similar expression pattern, and the accumulation pattern of flavonoid metabolites in the six varieties of figs varied, so we further analyzed the differences in the accumulation of flavonoid metabolites among the different varieties.

### 3.2. Analysis of Differentially Expressed Metabolites

**Figure 2:**
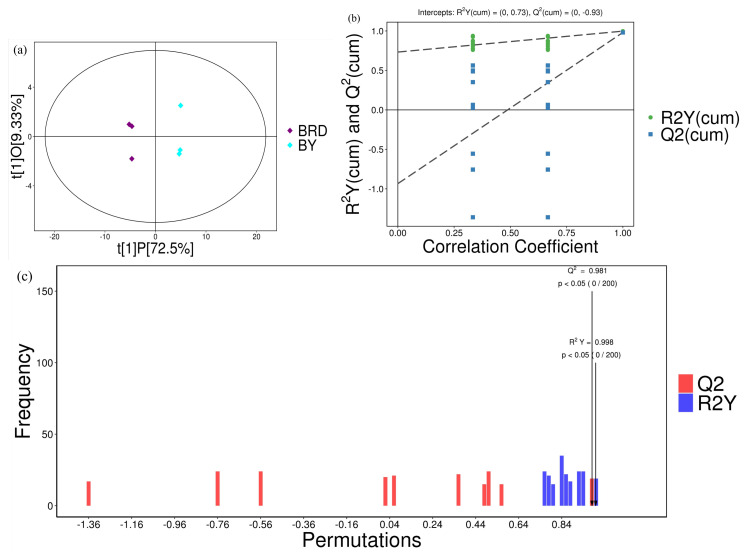
OPLS-DA model results and permutational test for the BRD vs. BY control group: spread plot of OPLS-DA results (**a**), OPLS-DA permutational test results (**b**), and bar chart of OPLS-DA permutational test results (**c**). Q2 is an important parameter for evaluating the OPLS-DA model, and R2Y denotes the percentage of information in the Y matrix that can be explained by the OPLS-DA model.

Multivariate and univariate statistics were implemented to identify differentially expressed metabolites in each control group. The model was the optimal model, as evidenced by the results of the OPLS-DA and the permutation test (Figure 2, Table S3). The VIP value in the OPLS-DA model was used to identify metabolites that were differentially expressed (VIP was greater than 0, and the *p*-value was less than 1). Table S4 displays a table of controls for each group, and 39 differentially expressed metabolites were screened for the 15 control groups. The MLY vs. BY group showed the maximum number of differentially expressed metabolites, with 30 being higher in MLY. The number of differentially expressed metabolites was lowest in JZ compared to MLY, and only seven were more abundant in JZ.

**Figure 3:**
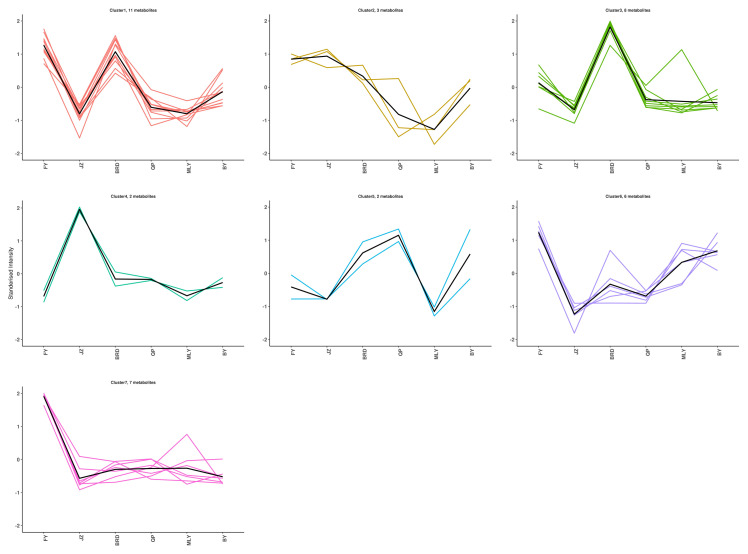
K-means analysis of 39 metabolites.

The 39 differential metabolites could be classified into seven enrichment patterns using K-means analysis (Figure 3, Table S5). BRD enriched eight flavonoid metabolites, primarily two flavanols, one dihydroflavonol, one flavonol glycoside, one dihydrochalcone, and one flavonol. JZ enriched two flavonoids, including one isoflavonoid and one Naringenin chalcone. Seven metabolites were enriched by FY, with one flavonol and one hydroxycinnamic acid being the most prominent.

**Figure 4:**
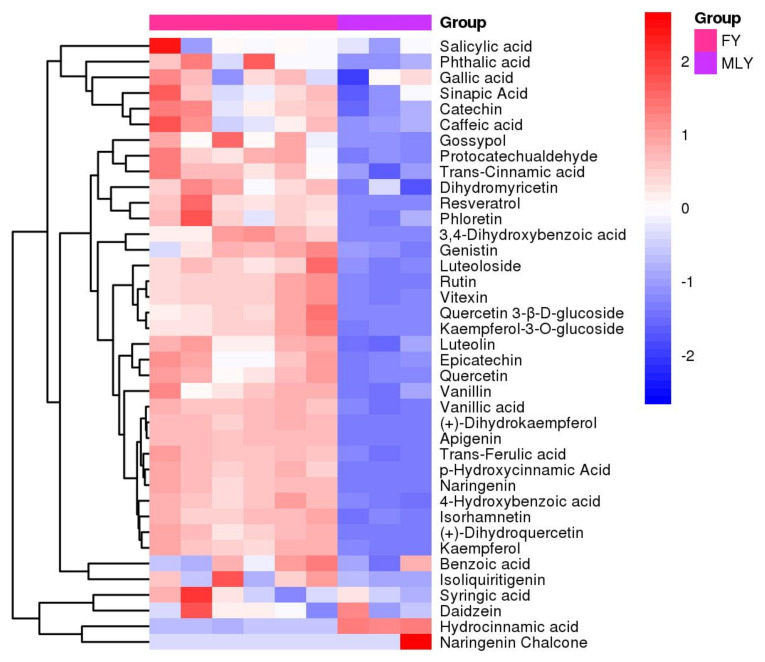
Heat map of metabolite hierarchical clustering in FY vs. MLY control group.

The hierarchical clustering heat map analysis revealed that 35 metabolites in the FY vs. MLY group exhibited elevated accumulation in FY (Figure 4). The 34 metabolites were highly accumulated in FY in the FY vs. JZ control group. In the BRD vs. MLY and BRD vs. JZ control groups, 32 and 33 metabolites were highly accumulated in BRD, respectively. The number of highly accumulated metabolites in the QP vs. MLY, QP vs. JZ, and BY vs. MLY, BY vs. JZ controls was greater in QP and BY than in MLY and JZ, but it was less in FY and BRD (S2). The six varieties exhibited a consistent pattern of high metabolite accumulation: black varieties accumulated more metabolites than green varieties, followed by yellow varieties.

Only 9 of the 39 metabolites were annotated to the flavonoid biosynthesis pathway, including four flavonols (C06562 Catechin, C09727 Epicatechin, C00389 Quercetin, C05903 Kaempferol), two flavonoids (C01514 Luteolin, C01477 Apigenin), two Dihy-droflavonols (C00774 Phloretin, C00509 Naringenin), one chalcone (C08650 Isoliqui-ritigenin), and five metabolites were annotated to the flavonoid and flavonol biosyn-thetic pathway (C01514 Luteolin, C05625 Rutin, C00389 Quercetin, C01477 Apig-enin, C05903 Kaempferol) (Table S6).

### 3.3. Transcriptomic Analysis

**Figure 5:**
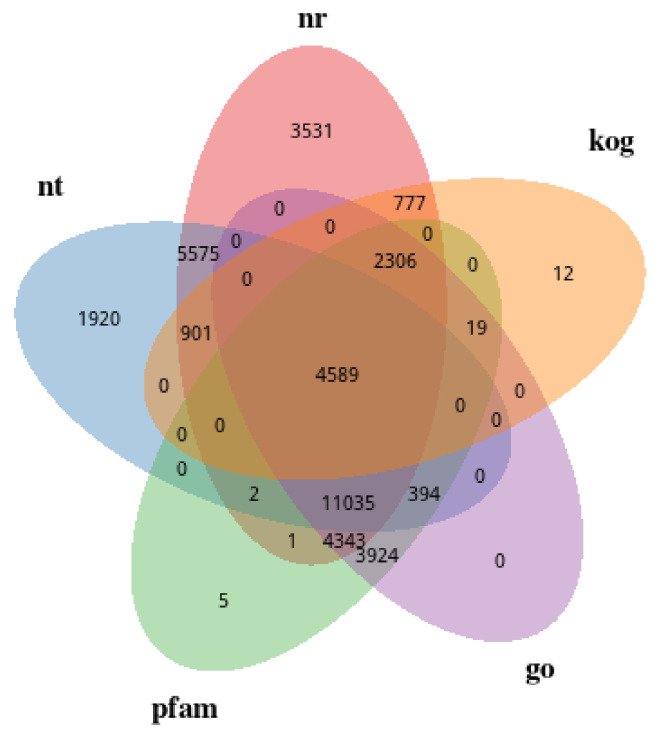
Venn diagram of gene annotation results.

To analyze the gene expression characteristics of fig MLY, JZ, BRD, FY, QP, and BY at the transcriptional level, 18 cDNA libraries were constructed and sequenced. The average Q30 and GC contents of the 18 libraries were 95.8% and 47%, respectively (Table S7). The 59,814 unigenes were assembled from a total of 2,611,016 clear reads: 33,060 (55.27%), 22,416 (40.81%), 26,618 (44.5%), 26,248 (43.88%), and 8604 (14.38%) (Figure 5, Table S8). A total of 62 genes belonging to the flavonoid biosynthesis pathway were identified, including F3H, CHS, CYP75B1, CYP73A, CYP98A, C3’H, DFR, FLS, and ANR (Table S9). Additionally, 1671 transcription factor genes were identified, including 68 genes in the WRKY transcription factor family, 44 bZIP, 74 bHLH, 130 MYB, and 86 genes in the AP2/ERF transcription factor family (Table S10).

### 3.4. Analysis of Differentially Expressed Genes

Analysis of variance revealed that a total of 20,136 differentially expressed genes (DEGs) were identified. The BRD vs. JZ control group had the highest number of differentially expressed genes, with 9317 genes, and the FY vs. BY control group had the lowest number, with only 3332 genes (Table S11). The gene expression in the flavonoid synthesis pathway varied among the varieties. For example, F3H, DFR, ANS, and FG2 were highly expressed in FY and BRD, while CYP75B1, ANR, and CHS were highly expressed in JZ (Table S12).

**Figure 6:**
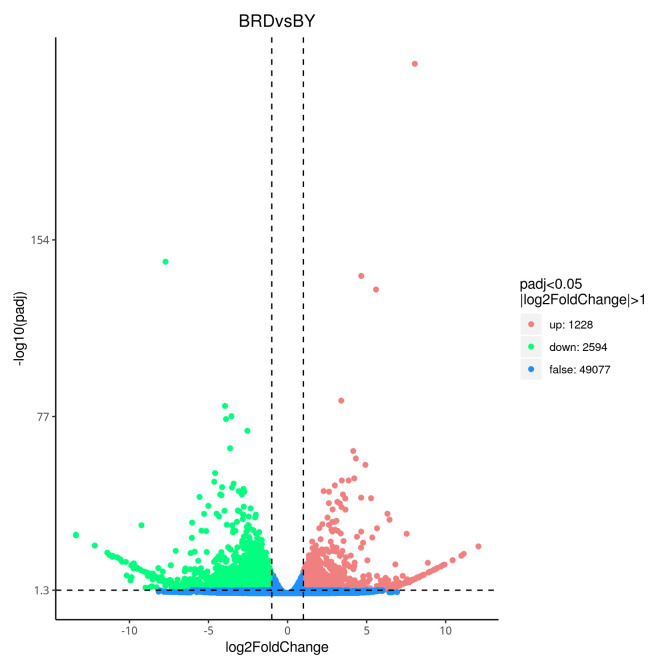
Differential gene volcano map.

Volcano plots were constructed for the differentially expressed genes in each group (Figure 6 and Figure S3), where a total of 3822 DEGs (1228 up-regulated and 2594 down-regulated) were identified in the BRDvsBY group, 9317 DEGs (3637 up-regulated and 5680 down-regulated) in the BRDvsJZ group, and 7660 DEGs in the BRDvsMLY group (3576 up-regulated, 4084 down-regulated); 4999 DEGs were identified in the BRDvsQP group (2382 up-regulated, 2617 down-regulated), 5278 DEGs were identified in the FYvsBRD group (2726 up-regulated, 2552 down-regulated), 3332 EDGs were identified in the FYvsBY group (1427 up-regulated and 1905 down-regulated), 7508 DEGs were identified in the FYvsJZ group (3045 up-regulated, 4463 down-regulated), 3507 DEGs were identified in the FYvsMLY group (1766 up-regulated, 1741 down-regulated), 4297 DEGs were identified in the FYvsQP group (2494 up-regulated, 1803 down-regulated). A total of 7403 DEGs were identified in the JZvsBY group (3871 up-regulated, 3532 down-regulated), 7106 DEGs were identified in the JZvsMLY group (4187 up-regulated, 2919 down-regulated), 7784 DEGs were identified in the JZvsQP group (4751 up-regulated, 3033 down-regulated), and 3439 DEGs were identified in the MLYvsBY group (4751 up-regulated, 3033 down-regulated). In the MLYvsQP group, 5111 DEGs were identified (2875 up-regulated, 2236 down-regulated), and 3904 DEGs were identified in the QPvsBY group (1578 up-regulated, 2326 down-regulated). In the MLY vs. BY group, there were a total of 3439 differentially expressed genes, of which 1578 were up-regulated and 2326 were down-regulated.

**Figure 7:**
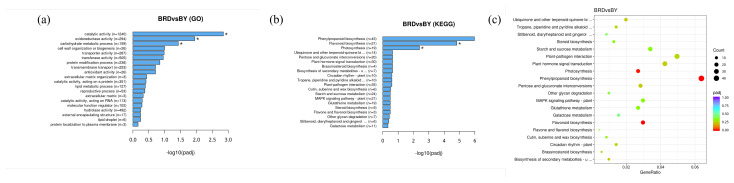
Gene sequence enrichment analysis. Asterisk means that there is a significant difference between this sample and a sample with a relative expression of 1. *, *p* < 0.05.

DEGs were enriched in three branches of the GO database: Molecular Function, Cellular Components, and Biological Processes, as demonstrated in Figure 7 and Figure S5. Among all controls, molecular function-enriched DEGs were the most numerous, and most of these genes were related to “Catalytic activity”, “Transferase function”, and “Oxidoreductase activity”. The most enriched cellular components were “Thylakoid”, “Cell wall”, and “Extracellular region”. The highest number of genes related to “Post-translational modification of proteins” and “Sugar metabolism in biological processes”.

**Figure 8:**
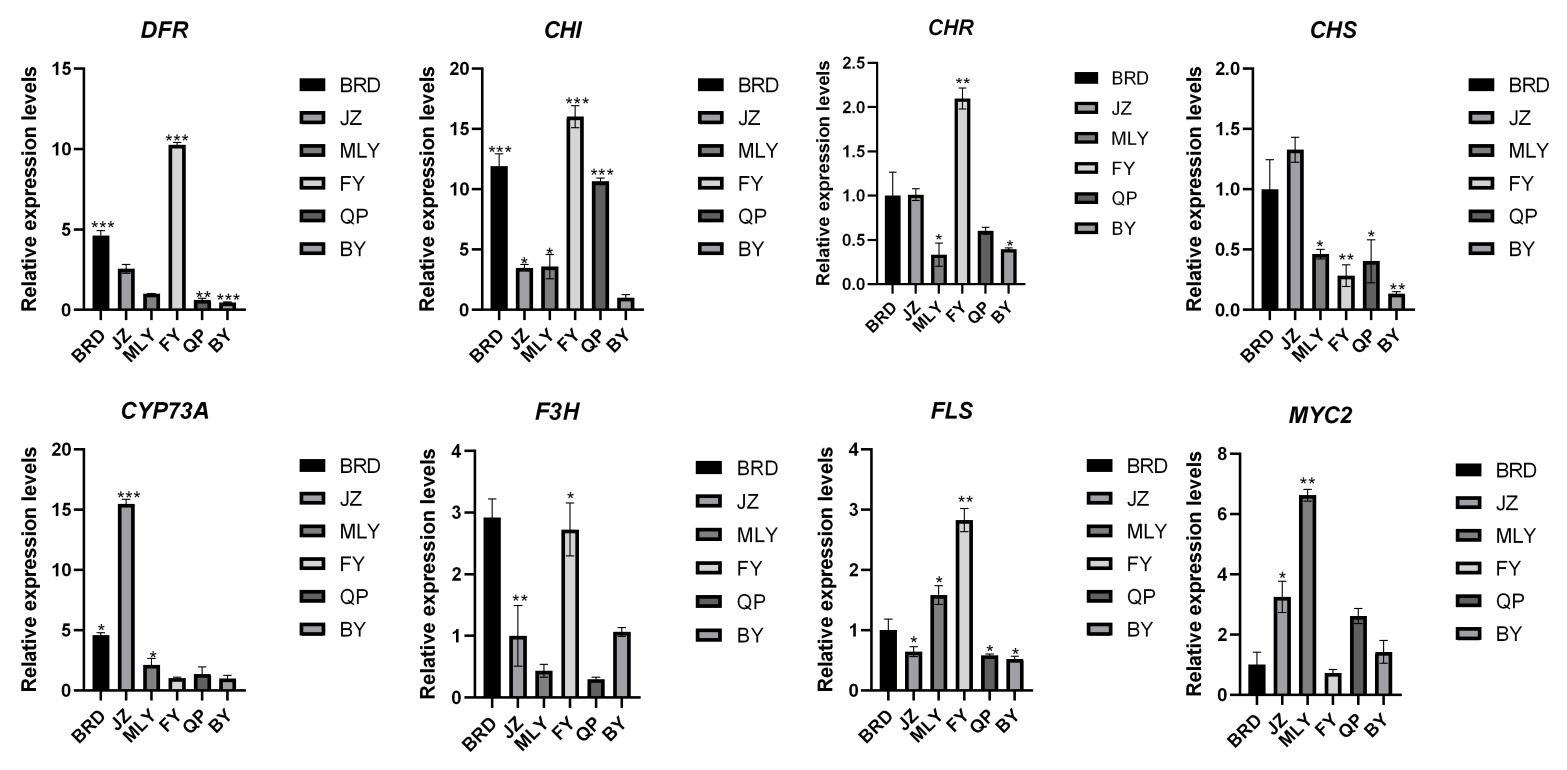
Relative expression levels of eight genes in six varieties of figs obtained by real-time fluorescent quantitative PCR (qRT-PCR) analysis. Asterisk means that there is a significant difference between this sample and a sample with a relative expression of 1. *, *p* < 0.05; **, *p* < 0.01; ***, *p* < 0.001.

KEGG metabolic pathways were annotated for DEGs to identify the metabolic pathways that are closely related among various varieties of fig fruits. All groups were ascribed to Flavonoid biosynthesis (ko00941), as demonstrated in Figure 7 and Figure S4, differently expressed genes annotated to the flavonoid biosynthesis (ko00941) metabolic pathway were present in all comparator groups, and there were also differentially expressed genes involved in the Flavone and flavanol biosynthesis (ko00944) metabolic pathway. The results showed that the metabolism of flavonoids varied among varieties with different degrees of accumulation due to gene expression. To further validate the reliability of the transcriptome results and gene expression levels, we selected eight flavonoid biosynthesis pathway genes for qRT-PCR analysis. The results showed that the expression profiles of the eight genes in the six fig varieties were consistent with the transcriptome sequencing results (Figure 8).

### 3.5. Flavonoid Biosynthetic Pathway Annotation and Analysis

To gain a more comprehensive understanding of the relationship between genes and metabolites, we conducted a more in-depth analysis of flavonoid synthesis pathway genes’ transcript and metabolite levels of flavonoid synthesis pathway genes in the fig transcriptome data. We identified a total of 10 DEMs that are annotated to the flavonoid synthesis pathway in six varieties of figs. These include two chalcone metabolites (Isoliquiritigenin and Naringenin Chalcone), one dihydrochalcone metabolite (Phloretin), four flavonol metabolites (Apigenin, Luteolin, Quercetin and Kaempferol), two flavanol metabolites (Catechin and Epicatechin) and trans-cinnamic acid. Based on the KEGG pathway annotation findings, 12 genes were identified in DEGs. These genes included 4-coumarate: coenzyme A ligase (4CL), Chalcone Synthase (CHS), Chalcone Isomerase (CHI), Cytochrome P450 (CYP73A, CYP75B1), Dihydroflavonol 4-reductase (DFR), Flavanone 3-Hydroxylase (F3H), Anthocyanidin Synthase (ANS), Anthocyanidin Reductase (ANR), Leucoanthocyanidin Reductase (LAR), and hikimate-O-hydroxycinnamoyltransferase (HCT). Most genes carried homologous genes, indicating a complex synthetic pathway for fig flavonoid metabolites.

**Figure 9:**
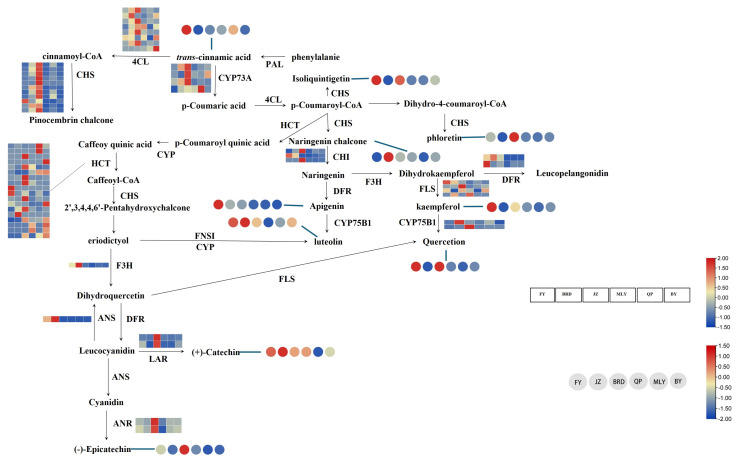
Heat map of fig flavonoid synthesis pathway genes and metabolites. The square heat map indicates the expression levels of key enzyme genes that are differentially expressed in different varieties. Circle heatmaps indicate the accumulation of different flavonoid metabolites in different varieties.

The transcriptome and metabolome data were integrated to create a heat map of genes and metabolites of the fig flavonoid synthesis pathway, as illustrated in Figure 9. This was done following the extant flavonoid synthesis pathway. The results indicated that the synthesis genes of the flavonoid pathway were most highly expressed in JZ, followed by BRD and FY. In-depth study revealed that *Fc4CL* was highly expressed in all varieties, *FcCYP73A* and FcCHS were highly expressed in JZ and BRD, *FcANR* was highly expressed in JZ, BY, and QP. *FcFLS* was highly expressed in BY in addition to JZ and BRD, and in MLY, none of the other genes except for *Fc4CL* were highly expressed. There was no significant difference in the expression of *FcHCT* in each variety. The metabolites were analyzed using a heat map, and the results indicated that Isoliquiritigenin, Kaempferol, Phloretin, Quercetin, and Epicatechin were highest in FY and BRD. Trans-cinnamic acid was highly expressed in FY, followed by MLY. Luteolin was primarily highest in FY and JZ, followed by BRD and BY. Naringenin Chalcone was mainly found in the JZ, followed by BRD, QP, and BY. Apigenin was highest in FY and lower in other species. JZ had the highest Catechin concentration, followed by FY, BRD, and QP.

The combined results indicated that flavonoids (Apigenin and Luteolin) and flavonols (Quercetin and Kaempferol) were the most abundant in FY. These metabolites were mainly regulated by genes such as *FcCHS*, *FcCHI*, *FcFLS*, *FcCYP*, and *FcDFR*, which were highly expressed in FY. The *FcCHS* gene was the primary regulator of Naringenin Chalcone, the most abundant metabolite in JZ, and was highly expressed. Conversely, the high expression of *FcHCT* and its homologous genes in all varieties suggests that a shared regulatory network may regulate the *FcHCT* gene. This pattern of high expression across varieties may indicate the existence of a general transcriptional regulatory mechanism that is conserved across species or varieties.

## 4. Discussion

Flavonoids are abundant in plants and can assist in reducing cellular damage, enhancing antioxidant activity, and promoting photosynthesis, among other functions [30]. They also possess significant pharmacological properties in anticancer [31], antimicrobial [32,33], and anti-inflammatory properties [34]. Currently, most research on figs is concentrated on the bioactivity of fig leaf and fruit extracts. However, the synthesis of fig fruit flavonoids and the potential mechanisms that regulate the synthesis of flavonoids are not well understood. Metabolomics analyses were conducted on 6 fig cultivars: QP, MLY, FY, BY, JZ, and BRD in this investigation. The results indicated that nine flavonoids were identified among 40 metabolites, a number lower than the 12 flavonoids reported in female flower tissues in the literature [35]. This may be attributable to the flavonoid-targeted metabolome’s restriction on the number of metabolites it can identify. The two black varieties ‘FY and BRD’ exhibited the highest accumulation of flavonoids in figs, as indicated by cluster analysis [36]. The high accumulation of phenolic compounds such as epicatechin and Quercetin 3-β-D-glucoside is consistent with the fact that epicatechin and Quercetin 3-β-D-glucoside are the main phenolic acids and flavonoids in fresh and dried figs and that the antioxidant capacity of figs is highly correlated with the content of phenolic compounds [37] . This may be attributed to flavonoids being a major component of color formation. The black varieties also contained the flavonoids Kaempferol-3-O-glucoside and Quercetin 3-β-D-glucoside, associated with color formation. This is corroborated by the discovery that the two flavonoid glycosides, Kaempferol-3-O-glucoside and Quercetin 3-β-D-glucoside, which are associated with color formation, were found to be highly accumulated exclusively in ‘FY and BRD’ [38]. The variance analysis revealed that metabolite accumulation varied substantially among varieties, particularly those with varying colors. The smallest difference was observed between varieties with the same color. The slightest differences were observed between varieties of the same color, while the largest differences were observed between black and yellow varieties. Our findings indicate that black varieties of figs may be more advantageous for pharmacological research.

Flavonoid synthesis in plants is primarily regulated by two categories of genes: genes that directly encode flavonoid synthesis-related enzymes and transcription factor genes that regulate these structural enzymes [39]. A total of 62 genes for flavonoid biosynthetic pathways were identified in our study, which analyzed six varieties of figs using transcriptomics. A total of 62 genes of the flavonoid biosynthesis pathway, including F3H, CHS, CYP75B1, CYP73A, CYP98A, C3’H, DFR, FLS, and ANR, and 1671 transcription factor genes were identified.

The phenylpropane biosynthetic pathway is the starting point for the biosynthesis of plant flavonoids. PAL, an enzyme that facilitates the transfer of primary metabolism to secondary metabolism in plants [40], and 4CL are both key rate-limiting enzymes in the phenylpropane bio-synthetic pathway [41]. Additionally, 4CL is involved in the lignin synthesis pathway. The material foundation for subsequent biosynthesis is established by the stable expression of *Fc4CL* in six varieties of figs. In the plant flavonoid synthesis pathway, the DFR and FLS genes will collaborate to generate colorless anthocyanins and flavonols by utilizing dihydroflavonols as precursors. However, there is still a discrepancy between the two in terms of the mechanism of competition [42]. The abundant expression of *FcDRF* in FY and BRD implies that the *FcDFR* gene facilitates the accumulation of anthocyanin metabolites. The high expression of *FcANS* in FY and BRD further supports this hypothesis. A distal enzyme of the anthocyanin synthesis pathway, ANS catalyzes the conversion of colorless anthocyanins into anthocyanin glycosides and is crucial for the development of plant color [43]. The high expression of *FcLAR* in the yellow variety of Fig. JZ and the significant accumulation of metabolites of Catechins suggest a close relationship between *FcLAR* and Catechin synthesis. This may also be the reason for the change in fruit color of this variety from green to yellow during the maturing process. However, experimental validation is lacking, and future experiments will be designed to determine the regulatory role of *FcLAR* in flavonoid biosynthesis. This is consistent with the discovery that the expression level of *CsLAR* increases as the Catechin content in tea trees increases [44]. It has been demonstrated that the MBW protein complex, which comprises R2R3-MYB TFs, bHLH TFs, and WD40 TFs, is the primary regulator of flavonoid biosynthesis in plants [45,46,47]. The MYB TFs were the most frequently identified in the current study, followed by AP2/ERF TFs and bHLH TFs. No WD40 TFs were identified. Family genes, which imply that the TF family that primarily modulates flavonoid biosynthesis in figs may be MYB and bHLH. This is comparable to the outcome that the formation of petal regions in NW peonies is jointly facilitated by the positive regulation of anthocyanoside biosynthesis by MYB and bHLH transcription factors [48]. Three bHLH family genes and two MYB genes are involved in accumulating anthocyanosides in peach fruits [28]. In conclusion, we examined the composition of flavonoids in figs of various varieties using metabolomics and elucidated the compositional distinctions between varieties. We identified the genes involved in flavonoid synthesis in figs and elucidated the expression characteristics of each related gene across various varieties using transcriptomics. The findings of this investigation offer some assistance in investigating flavonoid biosynthesis in figs.

## 5. Conclusions

By integrating transcriptome and targeted metabolome analyses, the study systematically resolved for the first time the differences in the accumulation of flavonoids and their synthetic regulatory mechanisms in different fig varieties, filling a knowledge gap in the field of fig secondary metabolism research. It was found that dark peel varieties (FY and BRD) had higher flavonoid accumulation, and key genes such as FcCHS, FcCHI, FcFLS, FcCYP, and FcDFR, and the MYB and bHLH transcription factor families. This finding reveals the molecular association between fig fruit color formation and flavonoid metabolism. This study innovatively adopted a multi-omics joint analysis strategy, breaking through the limitations of single-omics studies and providing a methodological reference for resolving complex plant metabolic pathways. In addition, the identification and functional analysis of the key genes lay the foundation for the subsequent targeted regulation of flavonoid synthesis through gene editing or metabolic engineering, which is of great significance for the development of fig medicinal value and functional food. Future studies can further validate the functions of the candidate genes and their regulatory networks, promote the efficient use of fig resources, and provide a reference for secondary metabolism studies of other economic crops.

## Data Availability

Data are contained within the article and Supplementary Materials.

## Supplementary Materials

The following supporting information can be downloaded at: https://www.mdpi.com/article/10.3390/biology14020184/s1. S1. Primer sequence information of eight candidate genes for fluorescence quantitative PCR. S2. Heatmap of clustering across comparison groups. S3. Differential gene volcano maps for 15 comparison groups. S4. Gene sequence GO enrichment analysis of 15 comparator groups. S5. Differential KEGG enrichment analysis of 15 comparison groups. Table S1. Quantitative analysis of 40 compounds. Table S2. Model information. Table S3. Hierarchical clustering data matrix. Table S4. Statistical table of differentially expressed metabolites in each control group. Table S5. K-means analysis data matrix. Table S6. Annotation analysis of 39 compounds. Table S7. Q30 and GC content of cDNA libraries. Table S8. Annotation of unigenes in 5 databases. Table S9. Flavonoid biosynthetic pathway gene expression. Table S10. The 1671 transcription factor genes identified. Table S11. Up- and down-regulation of differentially expressed genes in each control group. Table S12. Expression of flavonoid synthesis pathway genes in 6 fig varieties.
