# Supplementary material for: Integrated Transcriptome and Targeted Metabolome for Resolving Flavonoid Biosynthesis in Figs (Ficus carica Linn.)
Source: Biology (Basel). 2025 Feb 11;14(2):184. doi: 10.3390/biology14020184 (PMC11852052; doi:10.3390/biology14020184)
Supplement: Supplementary file 1 [file biology-14-00184-s001.zip › S1.pdf]

S1.Primer sequence information of eight candidate genes for fluorescence quantitative PCR

Gene ID	Annotation	Forward and Reverse primer sequences	Annealing (°C)
Cluster-21200.16099	CHI	F:CACTTTTCTCATGTGGTGAA	53.74
		R:TAAAGAGAAAATCGAGCAGC	53.75
Cluster-21200.19677	CYP73A	F:GTCCTCATGAATCTCTCCTT	53.84
		R:TGGGAAGAGCTGATAACAAT	54.01
Cluster-21200.18173	F3H	F:GCAAGAAAATAGTGGATGCT	53.94
		R:ATTAGAGATGAGTGCGGAAT	53.93
Cluster-21200.20418	MYC2	F:GAGAATAACGCCATAGCTTC	54
		G:CCAACCAAAAACCACCTAAT	53.92
Cluster-21200.17210	CHR	F:CGCCAAATGAAACCTCTAAA	54.2
		R:ATGGGGAGTTCTATTTGGTT	53.85
Cluster-21200.26526	FLS	F:GATGACAAAGAACCATGAGG	53.98
		R:ACTTCTCTGTAATTGGGAGG	54.04
Cluster-21200.16784	CHS	F:CCATTCTTTGATGGCCTTAG	54.01
		R:ATTGGATGCTAGACAAGACA	54.04
Cluster-21200.13486	DFR	F:AGGCATGCTTTCATTATGTC	54.01
		R:ATTAAAGGTTGTACCGGAGT	53.96
