# Supplementary material for: Integrated Transcriptome and Targeted Metabolome for Resolving Flavonoid Biosynthesis in Figs (Ficus carica Linn.)
Source: Biology (Basel). 2025 Feb 11;14(2):184. doi: 10.3390/biology14020184 (PMC11852052; doi:10.3390/biology14020184)
Supplement: Supplementary file 1 [file biology-14-00184-s001.zip › Reference Standard Information.pdf]

Reference Standard Information

名称	英文名称	分子式	CAS号
山奈酚	Kaempferol	C15H10O6	520-18-3
牡荆素	Vitexin	C21H20O10	3681-93-4
芹菜素	Apigenin	C15H10O5	520-36-5
二氢槲皮素	(+)-Dihydroquercetin	C15H12O7	480-18-2
二氢山奈酚	(+)-Dihydrokaempferol	C15H12O6	480-20-6
槲皮素	Quercetin	C15H10O7	117-39-5
芦丁	Rutin	C27H30O16	153-18-4
木犀草素	Luteolin	C15H10O6	491-70-3
柚皮素	Naringenin	C15H12O5	480-41-1
柚皮苷查尔酮	Naringenin Chalcone	C15H12O5	73692-50-9
儿茶素	Catechin	C15H14O6	7295-85-4
表儿茶素	Epicatechin	C15H14O6	490-46-0
二氢杨梅素	Dihydromyricetin	C15H12O8	27200-12-0
异鼠李素	Isorhamnetin	C16H12O7	480-19-3
山奈酚–3–O–葡萄糖苷	Kaempferol-3-O-glucoside	C21H20O11	480-10-4
槲皮素–3–O–葡萄糖苷	Quercetin 3-β-D-glucoside	C21H20O12	21637-25-2
反式阿魏酸	Trans-Ferulic acid	C10H10O4	537-98-4
4-羟基苯甲酸	4-Hydroxybenzoic acid	C7H6O3	99-96-7
氢化肉桂酸	Hydrocinnamic acid	C9H10O2	501-52-0
反式肉桂酸	Trans-Cinnamic acid	C9H8O2	140-10-3
香草酸	Vanillic acid	C8H8O4	121-34-6
香草醛	Vanillin	C8H8O3	121-33-5
没食子酸	Gallic acid	C7H6O5	149-91-7
苯甲酸	Benzoic acid	C7H6O2	65-85-0
水杨酸	Salicylic acid	C7H6O3	69-72-7
芥子酸	Sinapic Acid	C11H12O5	530-59-6
丁香酸	Syringic acid	C9H10O5	530-57-4
丁香醛	Syringaldehyde	C9H10O4	134-96-3
原儿茶酸	3,4-Dihydroxybenzoic acid	C7H6O4	99-50-3
咖啡酸	Caffeic acid	C9H8O4	331-39-5
对香豆酸	p-Hydroxycinnamic Acid	C9H8O3	501-98-4

原儿茶醛	Protocatechualdehyde	C7H6O3	139-85-5
白藜芦醇	Resveratrol	C14H12O3	501-36-0
邻苯二甲酸	Phthalic Acid	C8H6O4	88-99-3
异甘草素	Isoliquiritigenin	C15H12O4	961-29-5
大豆甙元	daidzein	C15H10O4	486-66-8
根皮素	phloretin	C15H14O5	60-82-2
4',5,7-三羟异黄酮-7-糖苷	genistin	C21H20O10	529-59-9
木犀草苷	luteoloside	C21H20O11	5373-11-5
棉酚	Gossypol	C30H30O8	303-45-7

Note: Standards are mainly from sigma.
