# Supplementary figures and images for: Integrated Transcriptome and Targeted Metabolome for Resolving Flavonoid Biosynthesis in Figs (Ficus carica Linn.)
Source: Biology (Basel). 2025 Feb 11;14(2):184. doi: 10.3390/biology14020184 (PMC11852052; doi:10.3390/biology14020184)

## S5.Differential KEGG enrichment analysis of 15 comparison groups.

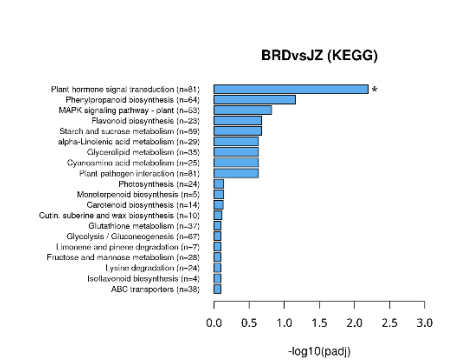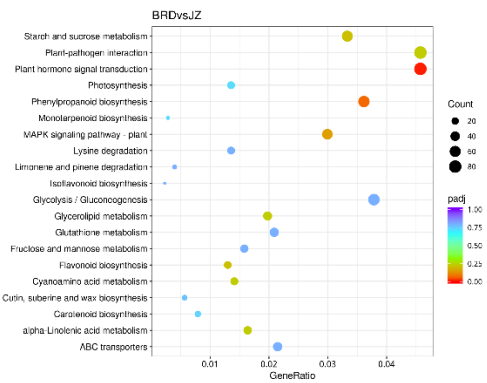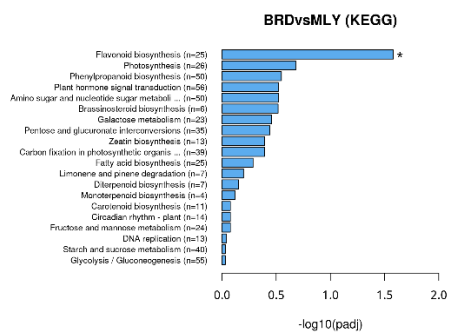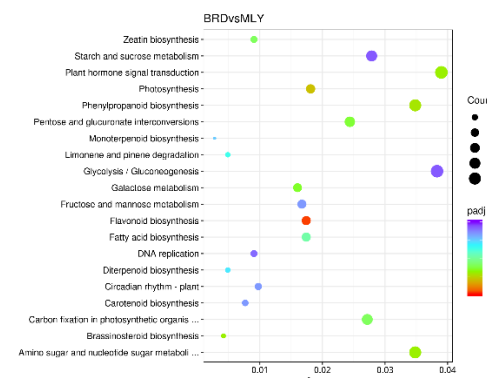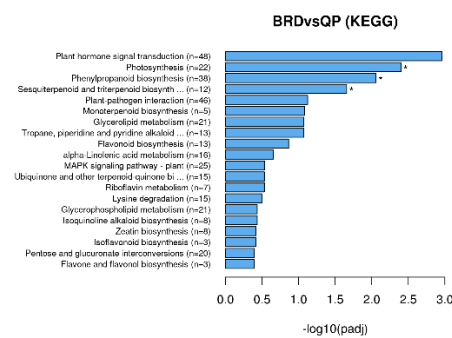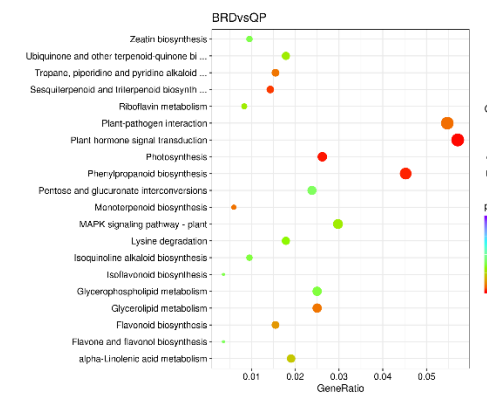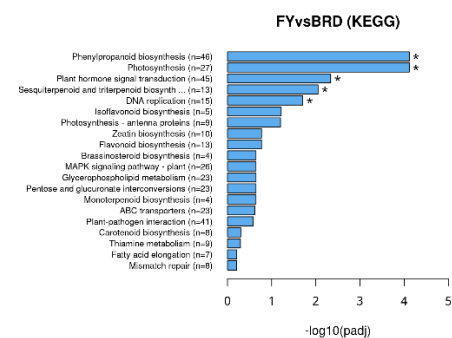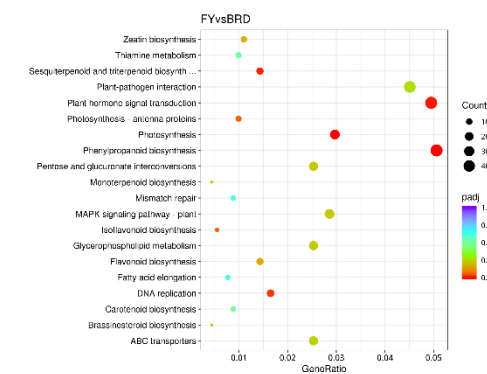

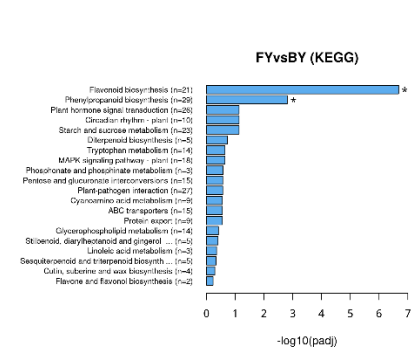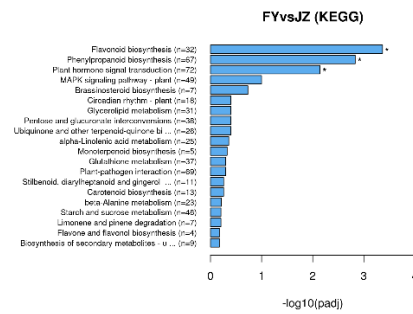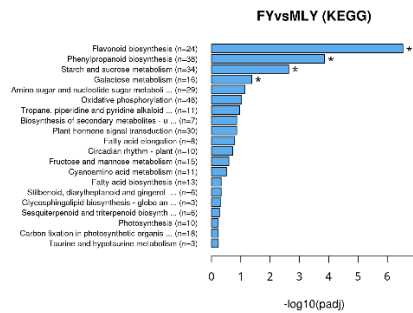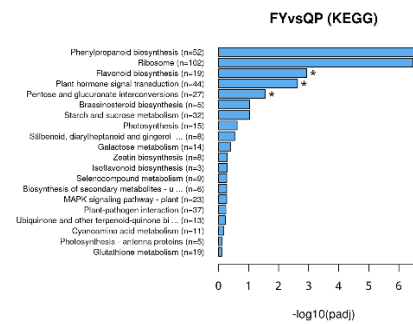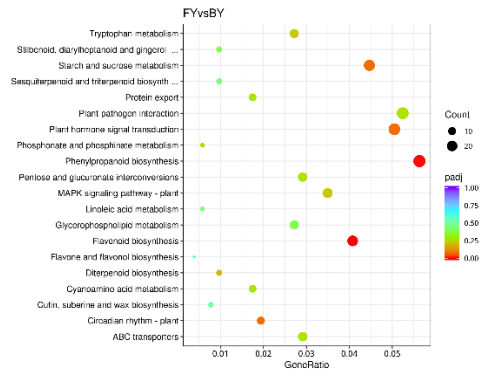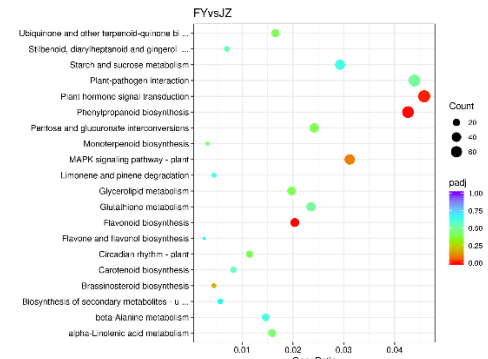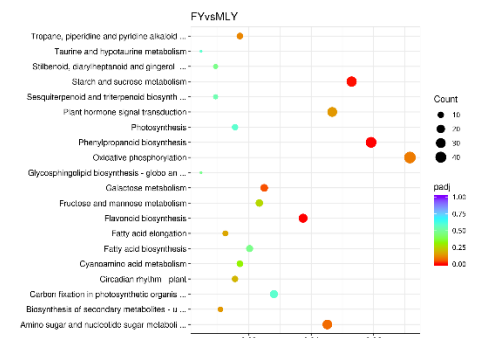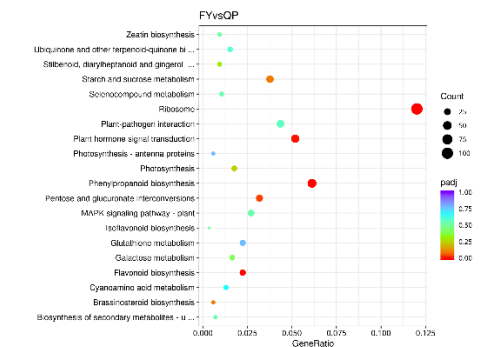

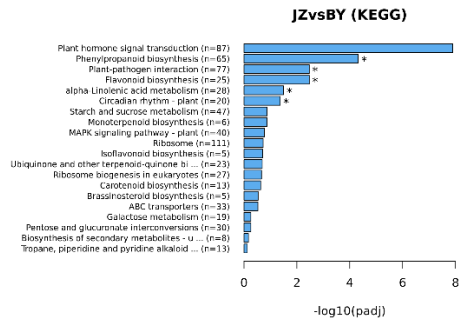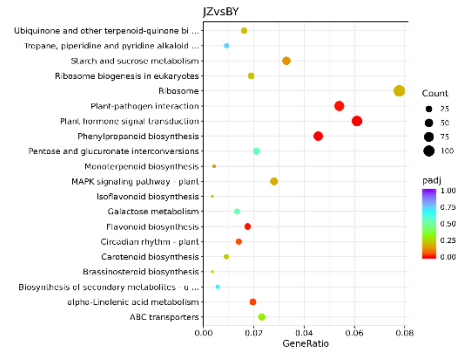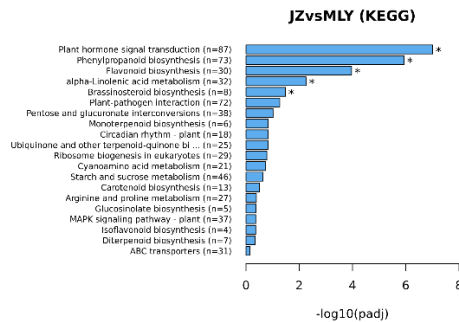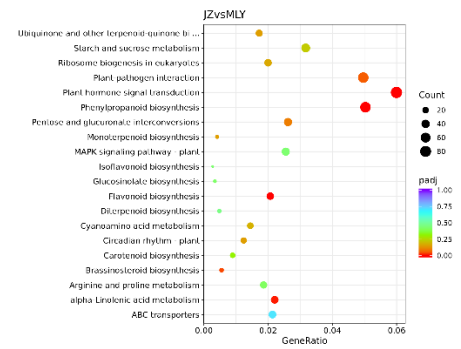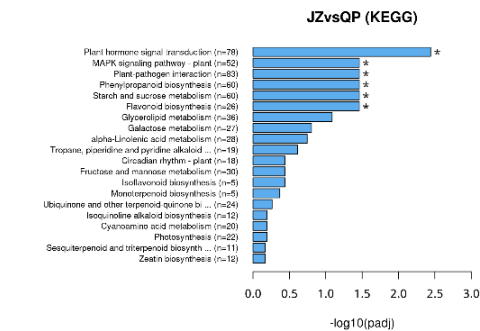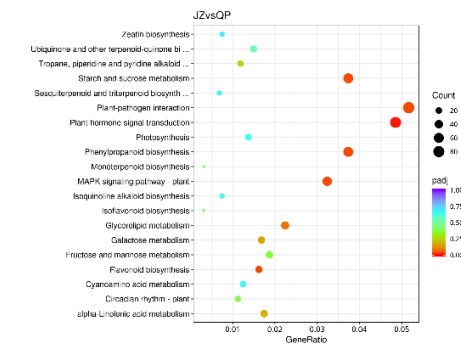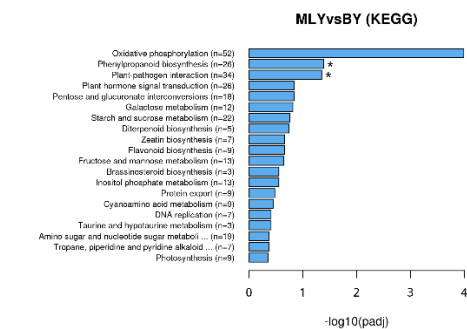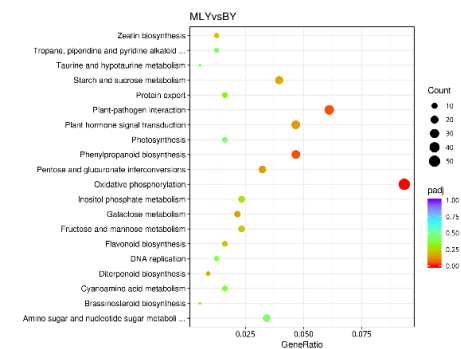

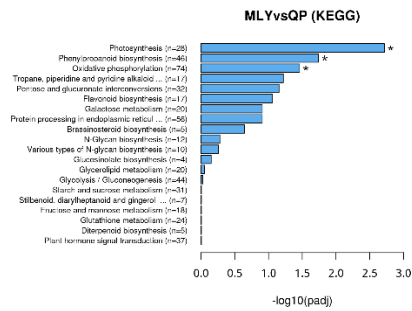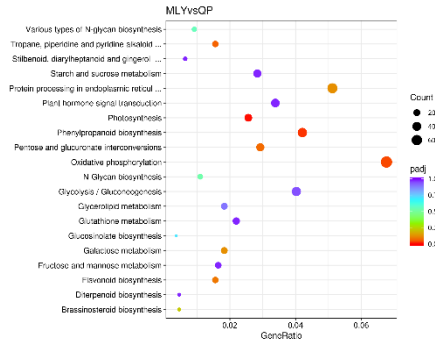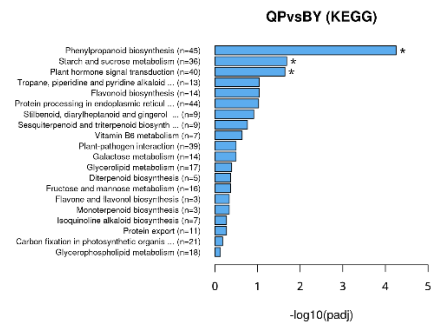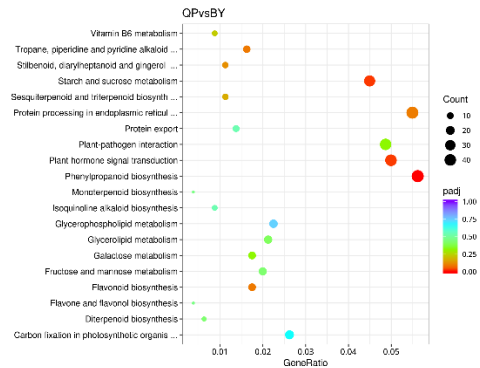

Supplement: Supplementary file 1 [file biology-14-00184-s001.zip › S5.pdf]

#### S4. Gene sequence GO enrichment analysis of 15 comparator groups.

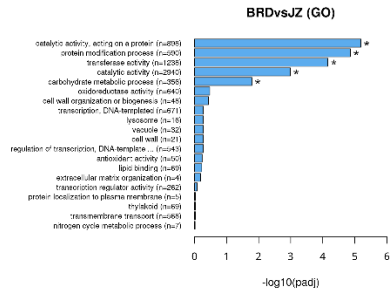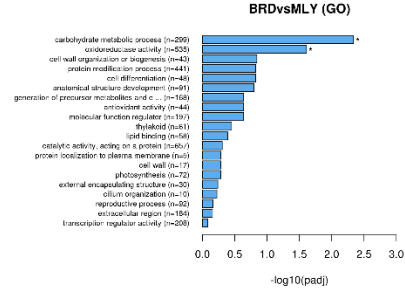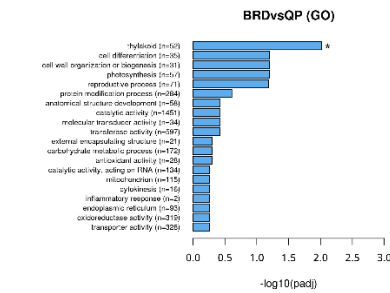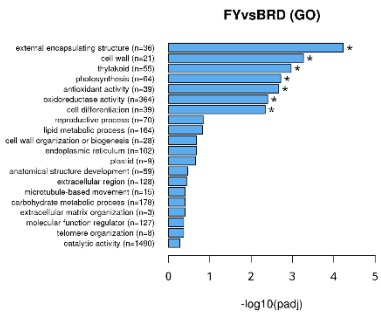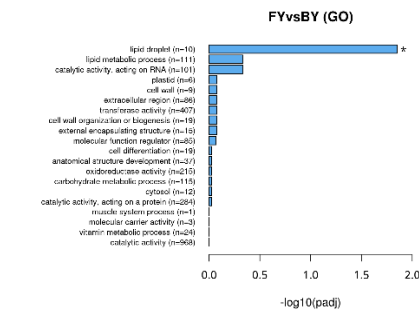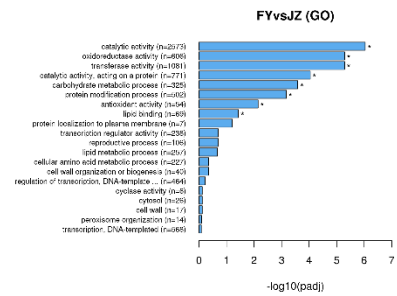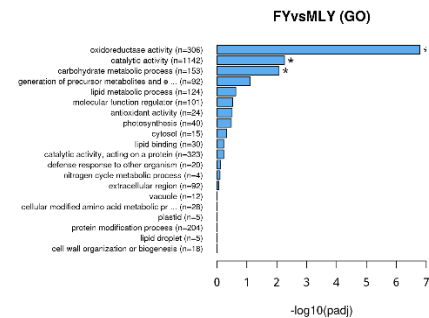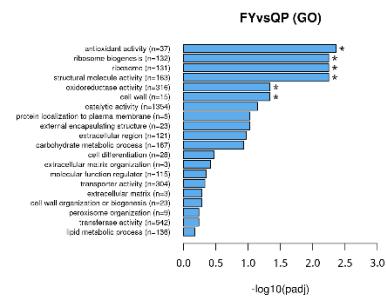

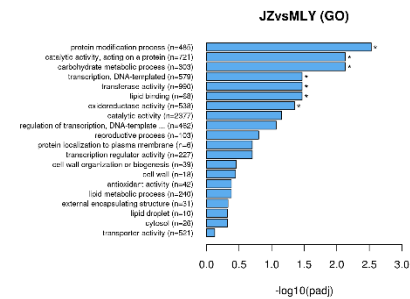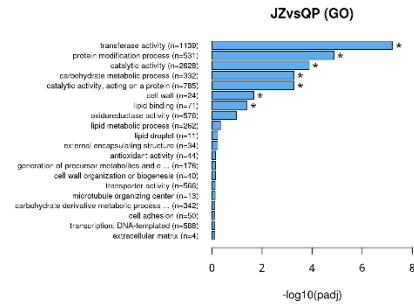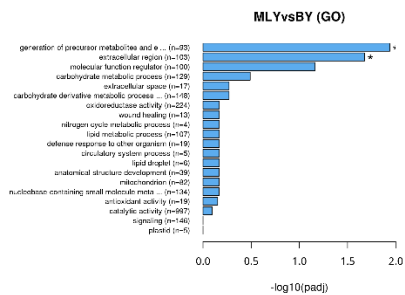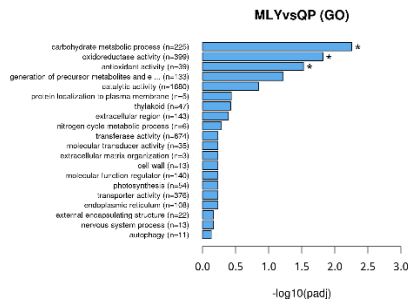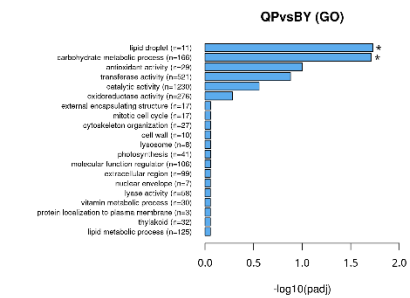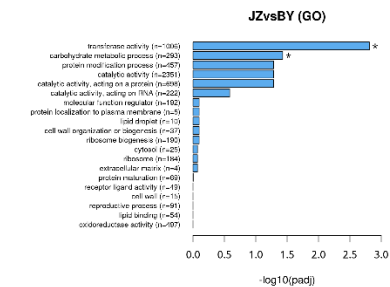

Supplement: Supplementary file 1 [file biology-14-00184-s001.zip › S4.pdf]

### S3.Differential gene volcano maps for 15 comparison groups.

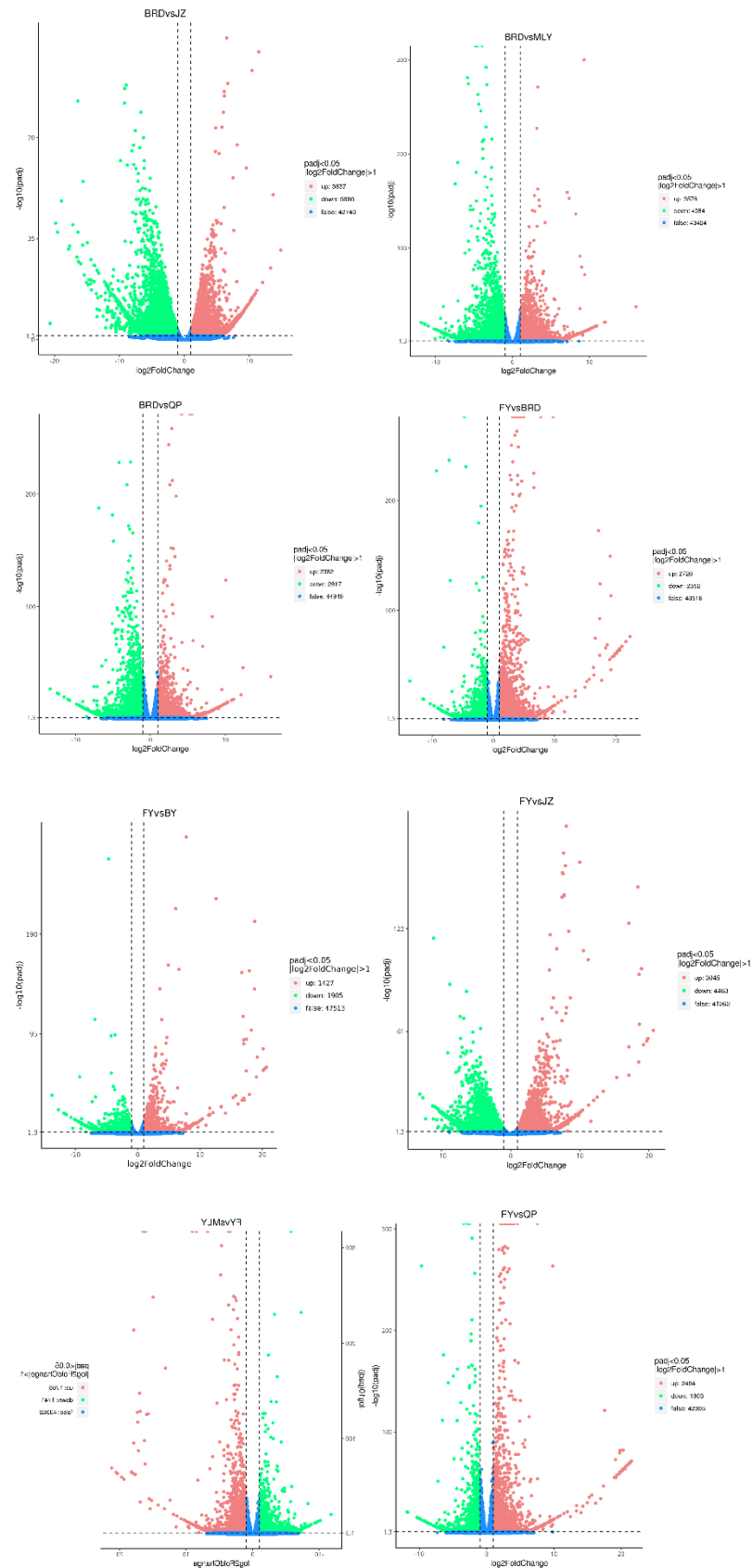

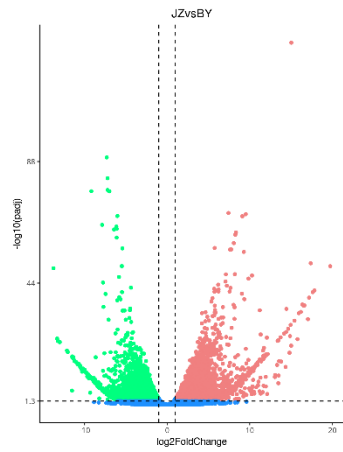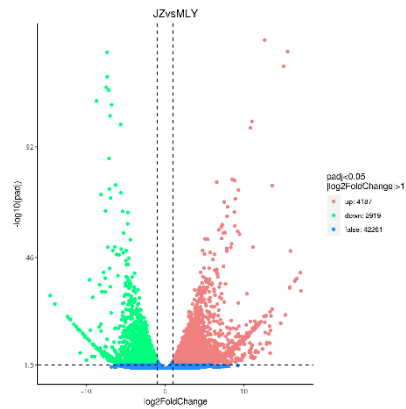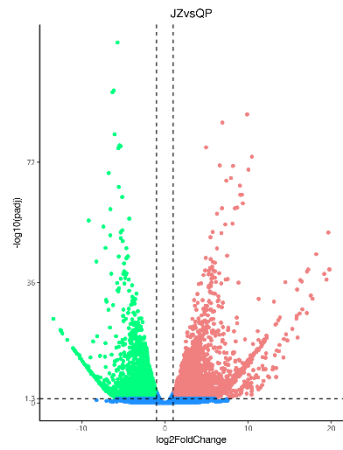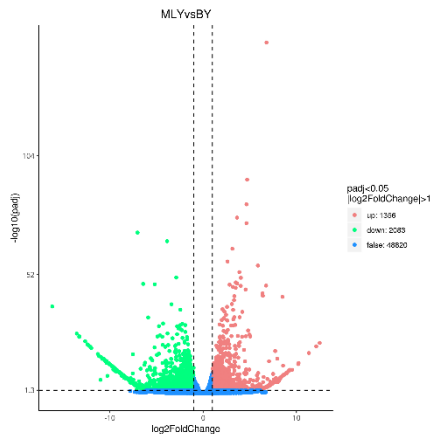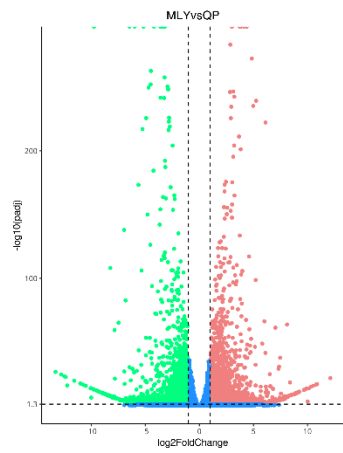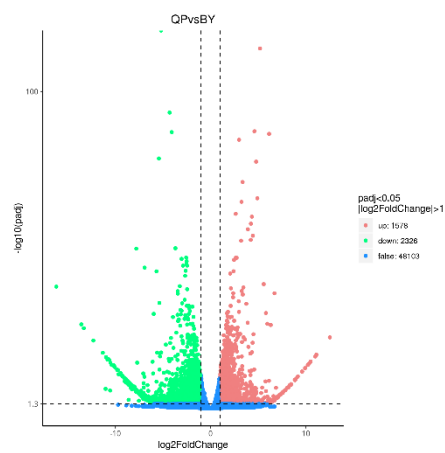

Supplement: Supplementary file 1 [file biology-14-00184-s001.zip › S3.pdf]

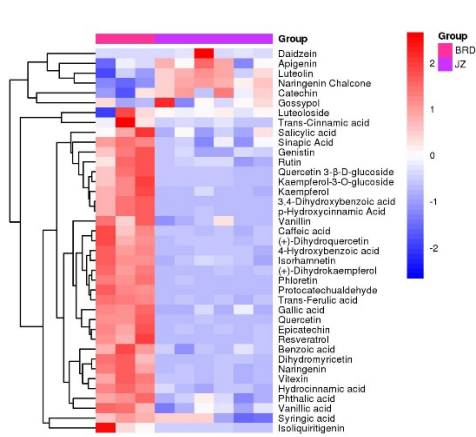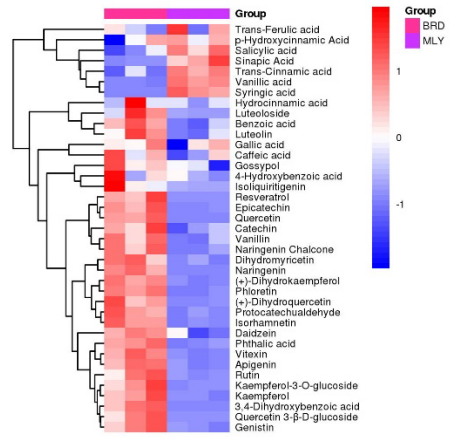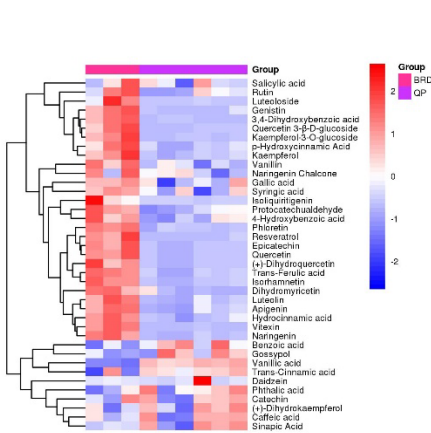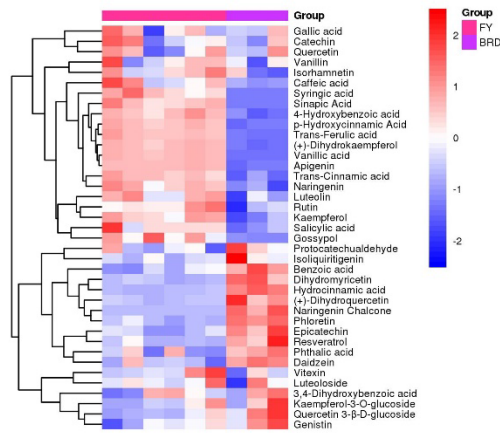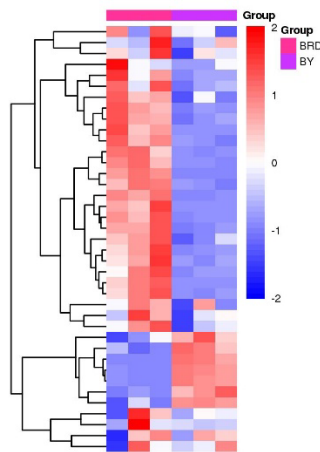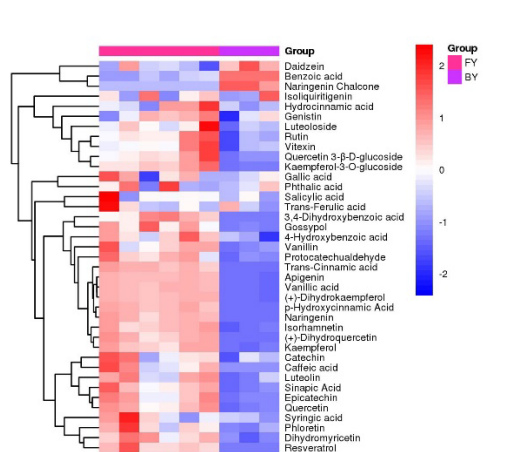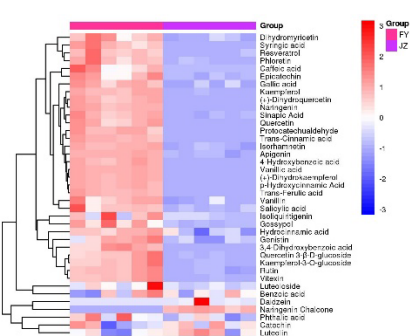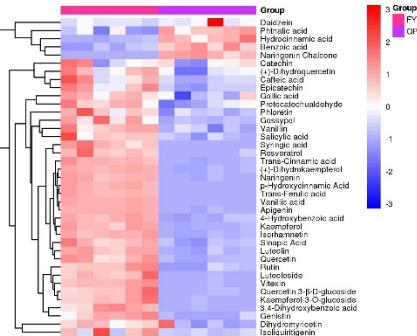

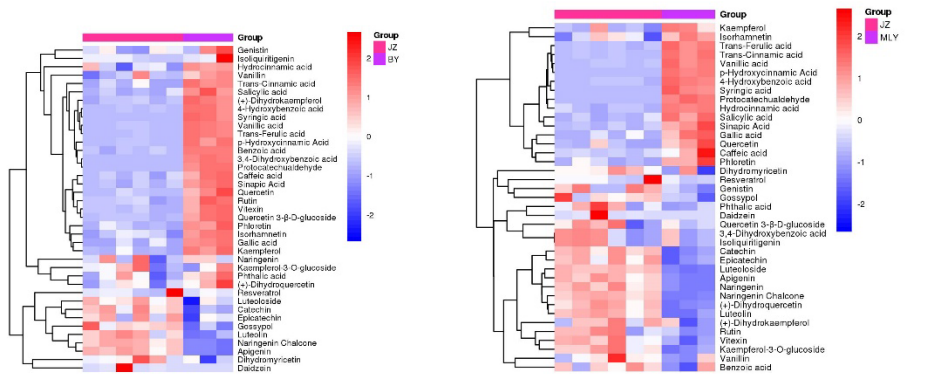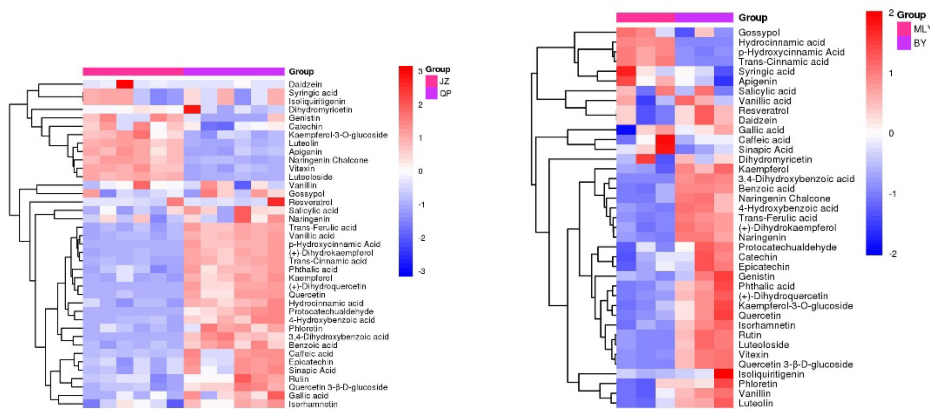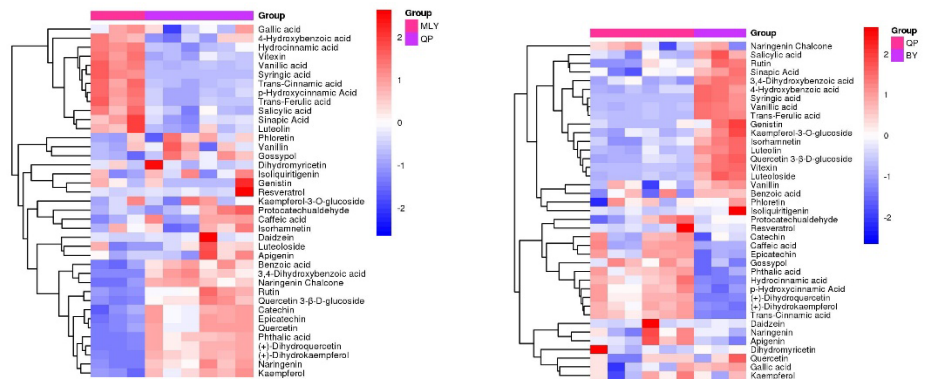

Supplement: Supplementary file 1 [file biology-14-00184-s001.zip › S2.pdf]
